# Obituary

**Published:** 1870-06

**Authors:** 


### OBITUARY.

DIED—In New Orleans, May 18th, Mrs. Lucie A. Roper, wife of Prof. John G. Angell, aged 23 years—eldest daughter of G. W. Roper, of that city.

Dr. Angell has the warm, heart-sympathy of all who knew him, in the irreparable loss he has sustained. In reference to the matter, we give place to the following action of the New Orleans Dental Society:

### IN MEMORIAM.

NEW ORLEANS, May 18, 1870.

At a special meeting of the New Orleans Dental Association, held this day, at the residence of Dr. Jas. S. Knapp, the following preamble and resolutions were adopted:

WHEREAS, It has pleased Divine Providence to take from a prominent member of our Association his beloved wife, and we desire to offer our condolence and sympathy to those who are most bereaved; be it

*Resolved*, That in the sudden and untimely death of Mrs. Dr. Jno. G. Angell, her husband, father, mother, son and other relatives have suffered a deep affliction and a loss which no human power can replace.

*Resolved*, That in the lovely and excellent character of the deceased she presented an example most worthy of imitation, and by her many virtues had won the highest esteem of all who knew her.

*Resolved*, That these resolutions be spread upon the minutes of the Association; that they be published in the city papers, and that a copy of the same be handed to the family of the deceased.

J. A. THURBER, President.

CHAS. L. THURBER, D.D.S., Recording Secretary.

